# Cerebral Correlates of Automatic Associations Towards Performance Enhancing Substances

**DOI:** 10.3389/fpsyg.2015.01923

**Published:** 2015-12-22

**Authors:** Sebastian Schindler, Wanja Wolff

**Affiliations:** ^1^Department of Psychology, Bielefeld UniversityBielefeld, Germany; ^2^Center of Excellence Cognitive Interaction Technology, Bielefeld UniversityBielefeld, Germany; ^3^Division of Sport and Exercise Psychology, University of PotsdamPotsdam, Germany

**Keywords:** EEG/ERP, anti-doping, attitudes, Implicit Association Test (IAT), indirect tests, substance abuse, Neuroenhancement (NE)

## Abstract

The direct assessment of explicit attitudes toward performance enhancing substances, for example Neuroenhancement or doping in sports, can be affected by social desirability biases and cheating attempts. According to Dual Process Theories of cognition, indirect measures like the Implicit Association Test (IAT) measure automatic associations toward a topic (as opposed to explicit attitudes measured by self-report measures). Such automatic associations are thought to occur rapidly and to evade voluntary control. However, whether or not such indirect tests actually reflect automatic associations is difficult to validate. Electroencephalography (EEG) has a superior time resolution which can differentiate between highly automatic compared to more elaborate processing stages. We therefore used EEG to examine on which processing stages cortical differences between negative or positive attitudes to doping occur, and whether or not these differences can be related to BIAT scores. We tested 42 university students (31 females, 24.43 ± 3.17 years old), who were requested to complete a brief doping IAT (BIAT) on attitudes toward doping. Cerebral activity during doping BIAT completion was assessed using high-density EEG. Behaviorally, participants D-scores exhibited negative attitudes toward doping, represented by faster reaction times in the doping + dislike pairing task. Event-related potentials (ERPs) revealed earliest effects between 200 and 300 ms. Here, a relatively larger occipital positivity was found for the doping + dislike pairing task. Further, in the LPP time range between 400 and 600 ms a larger late positive potential was found for the doping + dislike pairing task over central regions. These LPP amplitude differences were successfully predicting participants' BIAT D-scores. Results indicate that event-related potentials differentiate between positive and negative doping attitudes at stages of mid-latency. However, it seems that IAT scores can be predicted only by the later occurring LPP. Our study is the first to investigate the cerebral correlates that contribute to test scores obtained in the indirect testing of automatic associations toward doping. The implications of our results for the broader NE concept are discussed in light of the conceptual similarity of doping and NE.

## Introduction

Studies frequently find that considerable proportions of university students (Maier et al., 2013; Mazanov et al., 2013; Dietz et al., 2013a; Wolff et al., 2014), high-school students (McCabe et al., 2012) and traditional employees (Maher, 2008) use different types of substances to enhance their academic or work performance. This behavior has been labeled Neuroenhancement (NE; Wolff and Brand, 2013; Wolff et al., 2014). As NE is a relatively new research topic it has been suggested to draw upon knowledge from the conceptually similar, but theoretically and empirically much further developed field of doping research (Wolff and Brand, 2013). Semantically and conceptually, the similarity of both behaviors has been implied already (e.g., Maher, 2008; Dodge et al., 2012; Dietz et al., 2013b). The means-end relation represented by both behaviors is comparable. For example, erythropoietin (EPO) can be used in sports as a means to enhance athletic endurance (Lasne and de Ceaurriz, 2000). Among university students, Ritalin can be used as a means to enhance concentration (Forlini and Racine, 2009). Thus, both substances are used as means toward the end of performance enhancement. Social science research on doping is already much more evolved compared to research on NE and there already exists a wealth of knowledge on psychological processes that play a role in doping behavior (for a recent Meta-Analysis, see Ntoumanis et al., 2014). Among the best predictors of doping behavior are doping attitudes (Mallia et al., 2013; Ntoumanis et al., 2014). For the assessment of doping attitudes one needs to consider two different *types* of attitudes as explicated by Dual Process Theories of Cognition (Brand et al., 2015).

### Dual process theories of cognition

Dual-Process Theories of Cognition (e.g., Evans and Stanovich, 2013) postulate two different processes of thinking, resulting in two different types of attitudes (e.g., Gawronski and Bodenhausen, 2006). In the Associative-Propositional Evaluation Model (APE) Gawronski and Bodenhausen specify the features of associative (*implicit*) and propositional (*explicit*) attitudes: Associative processing is characterized by automatic affective reactions. This means that when presented with a doping stimulus, an athletes' affective association with doping is automatically activated (“doping is good”). This association does not have a truth value (i.e., it does not matter whether or not said athlete actually deems said association appropriate or inappropriate; for a doping specific overview please see Brand et al., 2015) and must not necessarily correspond to the results of propositional reasoning that characterizes explicit attitudes. Propositional reasoning is based on syllogistic inferences (“Doping is necessary to win”) that hence have truth values, meaning that they reflect a persons' reasoned evaluation toward a certain topic. There is ample evidence that shows that these different types of attitudes differentially affect behavior (e.g., Hofmann et al., 2005, 2007).

### Dual processes in doping research

Most research on social-cognitive predictors of doping so far has focused on the more traditional explicit attitudes (e.g., Ntoumanis et al., 2014). However, recent years have seen an increased focus on implicit doping attitudes (e.g., Petróczi et al., 2011; Brand et al., 2014b; Wolff et al., 2015). There are strong theoretical claims that the use of performance enhancing substances is not an entirely reflective process (e.g., Brand et al., 2015). Another reason is a measurement issue: Depending on what type of attitude one wants to assess, the ideal methods of assessment differ (e.g., Brand et al., 2015). Implicit attitudes are mostly assessed via indirect reaction-time based tests, whereas explicit attitudes are assessed using direct tests (i.e., self-report measures). However, direct tests are prone to response distortion when socially sensitive topics are addressed (Tourangeau and Yan, 2007). The social desirability of doping has been shown to influence self-reported doping attitudes (Gucciardi et al., 2010). Methods for the indirect assessment of automatic attitudes are reportedly much more robust toward such deliberate response bias problems (Wolff et al., 2015).

### Indirect attitude measurement

Implicit Association Tests (IAT; Greenwald et al., 1998) are computerized reaction-time based tests. Most generally, participants are asked to categorize two concepts (one target and one evaluative) as fast as possible with the same response key on the computer's keyboard. The speed of categorization varies as a function of the semantic association of these concepts. Closely associated concepts (e.g., flowers + like) are categorized faster than disjunct concepts (e.g., insects + like). One of the IAT's most important features from a measurement perspective is its postulated potential to control for the social desirability bias by evading voluntary control and being rather robust toward deception attempts compared to direct tests (Kämpfe et al., 2009). Indeed, compared with questionnaires, IATs display higher predictive validity when socially sensitive constructs are measured (Greenwald et al., 2009). Recently, a shorter and comparably valid and reliable IAT variant has been introduced: The Brief IAT (BIAT) (Sriram and Greenwald, 2009). The doping BIAT investigated here has been found to be a valid predictor for positive biochemical doping test results (Brand et al., 2014b).

### EEG measuring automaticity

In sum, for the assessment of socially sensitive topics (like doping or NE) indirect measures seem to be more suitable than direct measures (e.g., Wolff et al., 2015). However, such evidence does not allow for conclusions as to whether or not IAT scores actually reflect automatic associations toward the target concept. This is crucial if one wants to understand the actual cognitive processes that are reflected in the doping BIAT score. One way to test if doping BIAT scores reflect automatic associations is electroencephalography: Electroencephalography has a superior time resolution which can determine differential processing of doping attitudes in terms of milliseconds. The use of event-related potentials (ERPs) allows to investigate such differential processing on highly automatic or more deliberate processing stages and to relate these differences to actual BIAT performance. In general components are divided into ones with early (e.g., N1) middle (P2) and long latencies (LPP), where earlier components are thought to reflect more automatic processing. The N1 is thought to be a marker of visual discrimination of stimuli (Vogel and Luck, 2000) and more sensitive to physical stimulus properties (Olofsson et al., 2008). But even the N1 can be modulated by emotional content (Pourtois et al., 2004) or task context (Schindler et al., 2014). Further, the N1 component as well as the following P2 component are influenced by visual attention (Hillyard et al., 1998; Luck et al., 2000; Codispoti et al., 2006). On the other hand, the late positive potential (LPP) is thought to be a marker of elaborate evaluation of the stimulus and is connected to memory encoding (Dolcos and Cabeza, 2002; Schupp et al., 2007).

### ERP studies on the IAT

So far, a few studies have investigated event-related potentials of IAT completion (He et al., 2009; Hurtado et al., 2009; Ibáñez et al., 2010; O'Toole and Barnes-Holmes, 2010; Williams and Themanson, 2011). However, considerable variability of the used stimuli, investigated samples and analysis approaches makes these findings difficult to generalize. For example, Ibáñez et al. (2010) investigated early components, while Hurtado et al. (2009) analyzed the later occurring amplitudes. In both papers attitudes of indigenous and non-indigenous participants toward both groups were investigated. Results showed a stronger processing of the *incongruent* condition for indigenous participants (in this case, non-indigenous + like) at the early N170 and partially at the LPP. On the other hand, more frequently larger LPP amplitudes are reported for the *congruent* condition (He et al., 2009; O'Toole and Barnes-Holmes, 2010; Williams and Themanson, 2011). Williams and Themanson (2011) for example found no early effects, but found a larger LPP for straight couples paired with positive stimuli compared to gay couples and positive stimuli (Williams and Themanson, 2011). The authors reasoned that this might reflect the emotional congruency between the target concept and the evaluative concepts (i.e., positive/like). Further, regarding the relationship between ERP differences and IAT scores, amplitude differences in the LPP time window seem to be more consistently correlated with IAT scores (He et al., 2009; Hurtado et al., 2009; Williams and Themanson, 2011), while for early components, correlations are either not reported (Ibáñez et al., 2010), or found to be insignificant (He et al., 2009).

### Hypotheses

Taken together, some ERP studies show IAT differences already at early processing stages while late effects are reported more consistently. At these later stages a larger LPP can be expected for the congruent condition (in our case doping and dislike). Further, these later differences seem to be related to IAT scores.

We aim to further investigate the mechanisms involved in completing a BIAT on performance enhancing substances. To this aim, a large sample of participants performed a brief BIAT while high-density EEG was recorded. We investigated if doping attitudes measured by a validated doping BIAT are differentially processed on early and middle perceptual (N1, P2), or at late processing stages (LPP). The empirical findings of early perceptual differences during an IAT are inconsistent. Thus, investigations of the occipital N1 and P2 component are exploratory. However, we expected to find LPP differences, more precisely, a larger LPP for negative doping attitudes. Finally, we tested the hypothesis that differences at the LPP would be related to IAT D-scores.

## Methods

### Participants

Forty-eight students were recruited at the University of Bielefeld. They gave written informed consent and received course credit for participation. For a high-density EEG study, this is a rather large sample, enabled by data collection in two consecutive studies which investigated effects of different faking strategies on the doping BIAT (Schindler et al., 2015b; Wolff et al., in prep.). Specifically, in these studies baseline BIAT scores were compared to subsequently faked BIAT scores. These studies did not investigate content or congruency effects of these baseline scores and were not aimed at investigating how a non-faked BIAT score is associated with electrophysiological markers. The study was conducted in accordance to the Declaration of Helsinki and was approved by the ethics review board at the University of Bielefeld. One participant was excluded due to a history of previous mental disorder, another due to a previous brain tumor, and six participants due to excessive artifacts, leaving 42 participants for final analysis. One participant was left-handed.

These 42 participants (31 females) were 24.43 years old on average (*SD* = 3.17, *Min* = 20, *Max* = 30). Screenings with the German version of the Beck Depression Inventory and the State Trait Anxiety Inventory (Spielberger et al., 1999; Beck et al., 2001) revealed neither clinically relevant depression (*M* = 4.25, *SD* = 3.46) nor anxiety scores (*M* = 30.00; *SD* = 3.60).

### BIAT completion

We used a validated picture-based doping BIAT (Brand et al., 2014a). In this BIAT, the combined task consists of the combined classification of the target categories *doping* vs. *health food* with the attribute categories *like* vs. *dislike.* Since *doping* is the focal concept in this BIAT (i.e., the concept of interest), *doping* is mapped on the same response key (in our case the “I” key on the keyboard) throughout the whole test and only the attributes are changed across blocks (Sriram and Greenwald, 2009). In block A, *doping* and the attribute *like* share the same response key. In block B, *doping* and *dislike* share the response key “I.” Before each block the stimulus pairings that have to be categorized with the “I” key are shown on an introductory screen to allow participants to familiarize themselves with the stimulus material (*doping* + *like* in block A, *doping* + *dislike* in block B). The category labels that are relevant for the respective block (*doping* + *like*, or *doping* + *dislike*) remain on the top and bottom of the screen throughout the categorization task, to ensure that participants are aware what stimuli are focal in the current block. The *doping* concept was represented by pictures of pills, ampoules, and syringes; the *health food* concept by apples, cereal, and vegetables; the *like* attribute by positive emoticons; and the *dislike* attribute by negative emoticons.

### EEG recording

Participants were seated in a dimly lit and sound attenuated room. Continuous EEG signals were recorded from 128 BioSemi active electrodes (www.biosemi.com) with a sampling rate of 2048 Hz. During recording, Cz was used as a reference electrode. Biosemi uses two separate electrodes as ground electrodes: First, a Common Mode Sense active electrode (CMS), and second, a Driven Right Leg passive electrode (DLR). All electrodes were placed according to the 10–20 system using an elastic head cap. Four additional electrodes (EOG) measured horizontal and vertical eye movement. These were placed at the outer canthi of the eyes and below the eyes.

EEG data was pre-processed using Brain Electrical Source Analysis package (BESA; www.besa.de). Offline, data was re-referenced to the average reference. To identify and correct eye-movement artifacts the automatic correction algorithm implemented in BESA was used (Ille et al., 2002). EEG data was filtered using a 0.1 Hz (6db/oct) forward filter and a 40 Hz (24db/oct) zero-phase filter. Filtered data were segmented from 100 ms before stimulus onset until 1000 ms after stimulus presentation. One hundred millisecond before stimulus onset was used for baseline correction. Automatic artifact detection implemented in BESA was used to eliminate remaining artifacts defined as trials exceeding a threshold of 120 μV. In the doping + like block 13.04% and in the doping + dislike block 14.58% of the trials were rejected as artifacts, with no differences between the blocks [*t*_(41)_ = 0.95, *p* = 0.35]. For both conditions about 34 trials were included for averaging.

### BIAT analyses

Behavioral data was analyzed with JASP (www.jasp-stats.org/, Love et al., 2015). Paired *t*-test were set-up to investigate differences in raw-reaction times as well as effects for the resulting *D*-scores between both blocks. *D*-scores are already a standardized aggregate measure of reaction time differences between the doping + like and the doping + dislike block. Effect sizes for repeated measures were calculated for all statistical tests (Cohen, 1988). Finally, parametric Pearson correlations were calculated between BIAT D-scores and mean microvolt amplitude differences of ERP components.

### EEG data analyses

EEG data were analyzed with EMEGS (http://www.emegs.org/, Peyk et al., 2011). For statistical analyses, paired *t*-tests were set-up to investigate differences between both blocks (block: doping + like vs. doping + dislike) in time windows and electrode clusters of interest. Effect sizes were calculated for all statistical tests (Cohen, 1988).

We investigated congruency effects on the N1, P2 and LPP components (see also Williams and Themanson, 2011). These ERP components were quantified post-stimulus for the occipital N1 from 150 to 200 ms and for the occipital P2 from 200 to 300 ms. Fronto-centrally, the LPP was identified from 400 to 600 ms. For the occipital sensor cluster 20 electrodes were investigated (PO7, PO9h, PO9, PO3, POO3, O1, OI1, I1, POOz, Oz, OIz, Iz, POO4, O2, OI2, I2, PO6, PO8, PO10h, and PO10) and over fronto-central locations a cluster of eighteen electrodes was used (FC1, FCz, FC2, FCC1, FCC1h, FCCz, FCC2h, FCC2, C3h, C1, C1h, Cz, C2h, C2, C4h, CCP1h, CCPz, and CCP2h).

## Results

### Behavioral results

Mean reaction times for the doping + dislike block (*M* = 688 ms, *SD* = 118 ms) were significantly faster than for the doping + like block [*M* = 790 ms, *SD* = 286 ms; *t*_(41)_ = 2.37, *p* < 0.05, *d* = 0.47]. Participants' average doping attitudes as measured by their D-Score in the BIAT displayed a significantly negative affect toward doping [*M* = −0.24, *SD* = 0.52; *t*_(41)_ = 3.00, *p* < 0.01, *d* = 0.94].

### EEG results

#### Occipital sensor cluster: N1 (150–200 ms) and P2 (200–300 ms)

**Figure 1:**
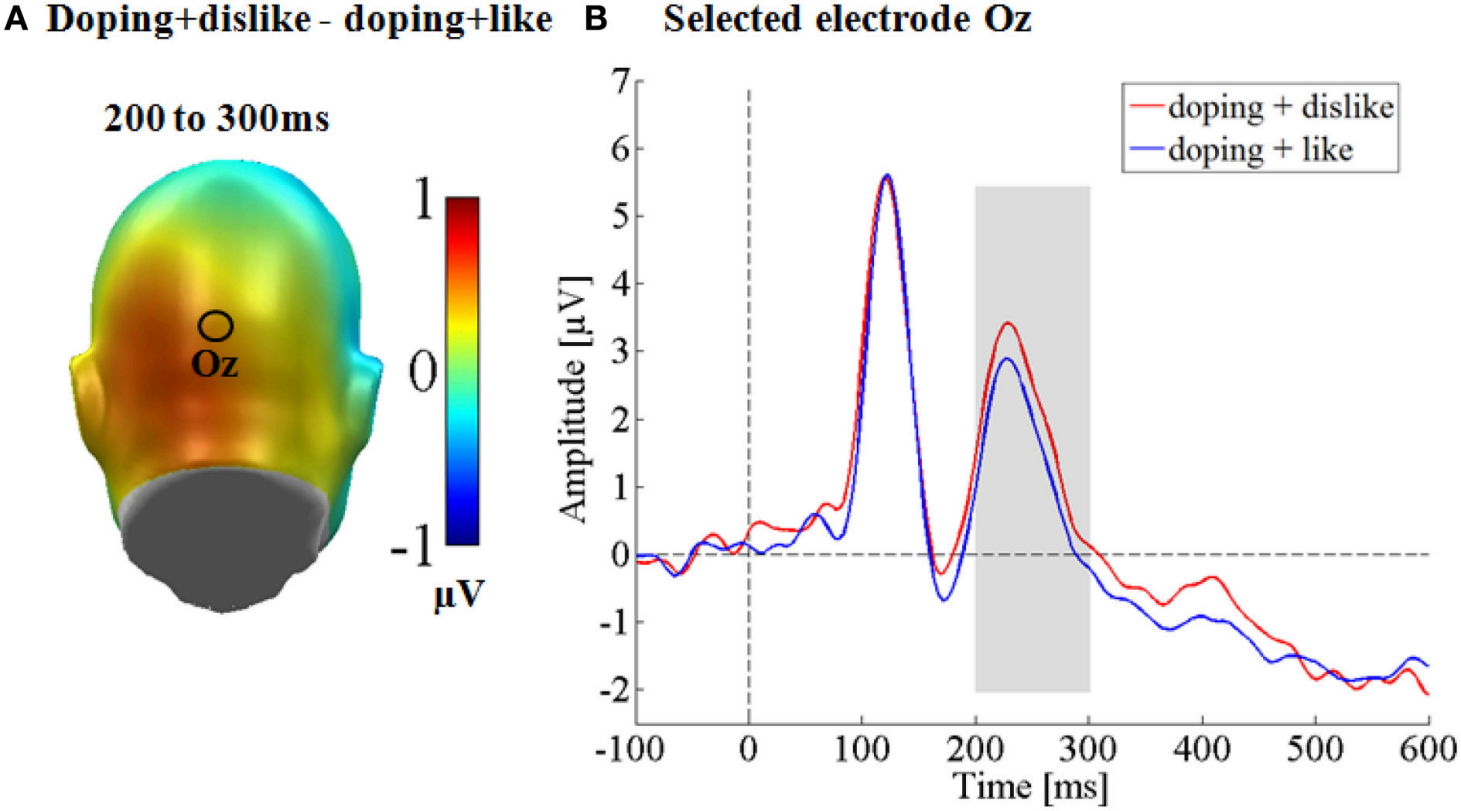
**Occipital effects on the N1 and P2 components. (A)** Difference topographies for negative doping affect minus positive doping affect **(B)** selected electrode Oz for the occipital electrode set, displaying the time course over occipital sites.

For the occipital N1 (150–200 ms), no significant differences were observed [*t*_(41)_ = 1.27, *p* = 0.21, *d* = 0.07]. At the occipital P2, significantly larger amplitudes were observed for the doping + dislike condition [*t*_(41)_ = 2.55, *p* < 0.05, *d* = 0.13; see Figure 1].

#### Fronto-central sensor cluster: LPP (400–600 ms)

**Figure 2:**
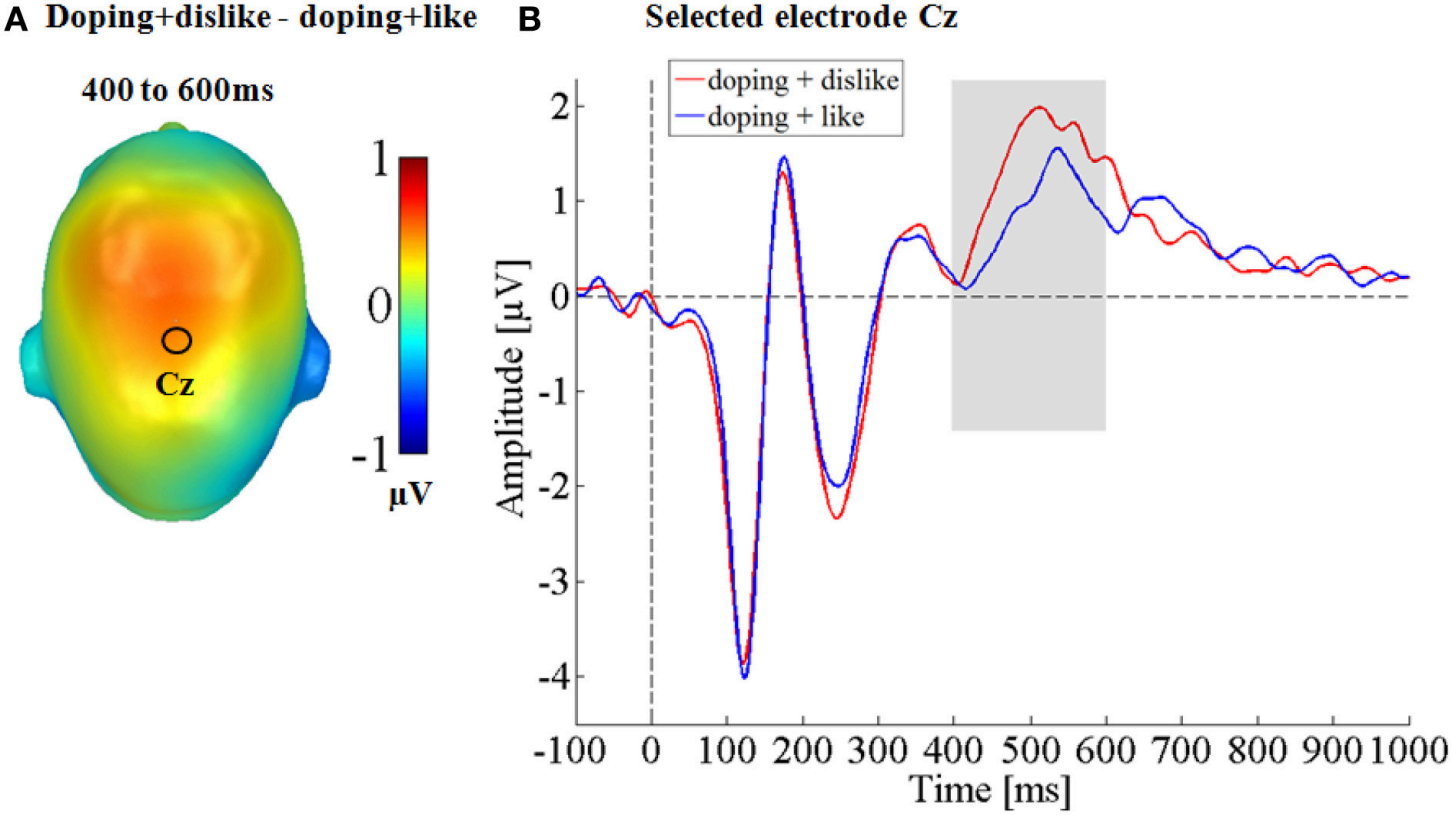
**Central effect on the LPP component. (A)** Difference topographies for negative doping affect minus positive doping affect **(B)** selected electrode Cz for the central electrode cluster, displaying the time course over central sites.

Over the central sensor cluster, in the time window of the Late Positive Potential a main effect of condition was found. Here, the doping + dislike block was found to elicited a significantly larger LPP compared to the doping + like block [*t*_(41)_ = 2.80, *p* < 0.01, *d* = 0.23; see Figure 2].

#### Relationship between ERPs and BIAT D-scores

**Figure 3:**
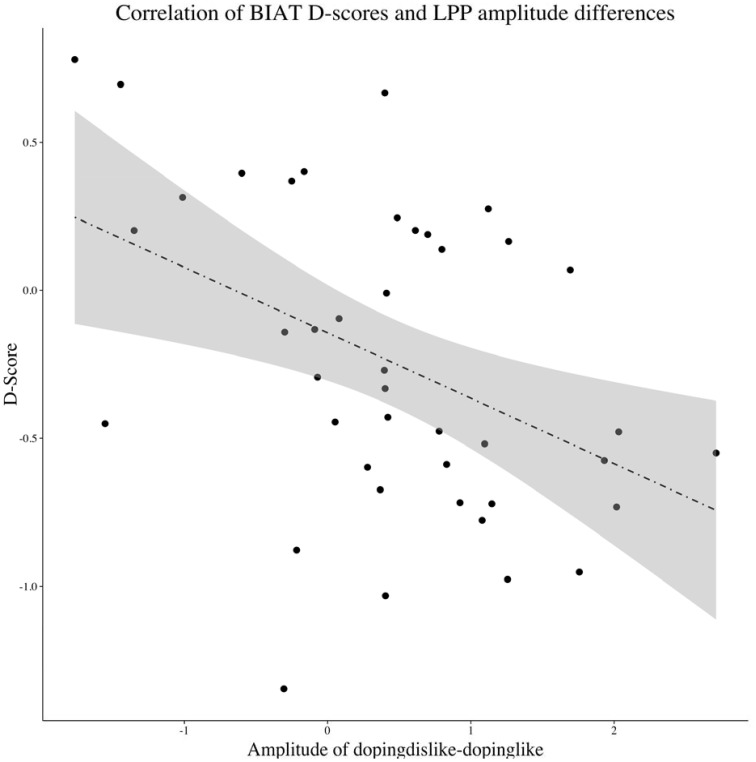
**Significant correlation (*r* = −0.43, *p* ≤ 0.01) between LPP amplitude differences and BIAT D-Scores**. The shaded area represents the standard error for each conditional mean as predicted by the dotted regression line.

For the occipital sensor cluster, no significant relationship between amplitude differences and BIAT D-scores were observed regarding the N1 or the P2. While significant differences in the processing could be observed on the occipital P2, these differences could not be related to the behavioral differences (*N* = 42, *r* = 0.18, *p* = 0.26). However, at the late processing stages of the LPP, amplitude differences were significantly correlated with BIAT D-scores (*N* = 42, *r* = −0.43, *p* < 0.01; see Figure 3). Here, with increasing anti-doping D-scores, the LPP amplitude differences became larger.

## Discussion

This study investigated the cerebral processing of negative and positive attitudes toward performance enhancing substances measured by a doping BIAT. As expected, the doping BIAT scores in our sample of university students reflected a negative attitude toward doping. Specifically, they were faster when doping and dislike shared the same response key and slower when doping and like shared the same response key. This resulted in a significant negative D-score. Further, neuroscientifically we found differences on middle and late ERP components: An enhanced occipital P2 as well as a larger LPP for negative doping attitudes.

We expected to find differences at late processing stages, namely an enlarged LPP for the congruent condition (i.e., the doping + dislike block), based on previous ERP reports (He et al., 2009; Hurtado et al., 2009; O'Toole and Barnes-Holmes, 2010; Williams and Themanson, 2011). Indeed, by examining attitudes toward performance enhancing substances we could observe a larger LPP for the congruent condition. Further, the larger LPP for negative doping attitudes was also linked to the BIAT D-score. Larger LPP effects for negative doping attitudes were found to co-occur with stronger anti-doping D-scores.

Larger LPPs have been previously found for emotional congruent target stimuli (Dillon et al., 2006; Spreckelmeyer et al., 2006). Thus, the larger LPP for the congruent IAT condition has been interpreted to reflect a form of emotional congruency (Williams and Themanson, 2011).

Typically, larger LPPs are also found for emotional compared to neutral pictures (Schupp et al., 2004b, 2007), interpreted in terms of facilitated attention toward these emotional stimuli (Cuthbert et al., 2000; Schupp et al., 2004a) due to their higher relevance for reproduction and survival (Lang et al., 1997). In this experiment the pictures and emoticons were the same in both conditions. However, research has demonstrated that the LPP can be modulated by giving instructions about stimulus interpretation (Hajcak and Nieuwenhuis, 2006; Foti and Hajcak, 2008). In the same vein, context effects have been shown to change ERPs toward physically identical stimuli in various paradigms (Bublatzky and Schupp, 2012; Fields and Kuperberg, 2012; Wieser and Keil, 2013; Schindler et al., 2014, 2015a; Klein et al., 2015). Here, larger LPP amplitudes typically occurred in the more salient context, e.g., in the condition were participants expect a threat of shock (Bublatzky and Schupp, 2012), which is more self-relevant (Fields and Kuperberg, 2012), emotionally more engaging (Klein et al., 2015) or socially more intense (Wieser and Keil, 2013; Schindler et al., 2015a). Similarly to explicit instruction-dependent LPP modulations (Foti and Hajcak, 2008; Hajcak and Nieuwenhuis, 2006), participants might implicitly perceived the doping and dislike combination to be emotionally more arousing or salient.

Further, regarding the early differences between both conditions we found a larger occipital P2 for negative doping attitudes. There is evidence that in visual paradigms the parieto-occipital P2 originates from extrastriate visual cortex (Mehta et al., 2000). Regarding its functional meaning, it has been found that the P2 is influenced by visual attention, where larger P2 components are observed for attended stimuli (Luck et al., 2000). Further, the P2 can be a predictor for subsequent visual detection success (Mathewson et al., 2009), where larger P2 components during fixation cross presentation were found for trials which were later detected compared to trials which were missed (Mathewson et al., 2009). It is unclear why these early differences emerged when responding to perceptually identical stimuli. It might be that participants were more engaged in the doping and dislike task and paid more attention to the stimuli. However, these findings need to be replicated, as early effects are not consistently found in ERP studies on the IAT (Williams and Themanson, 2011).

Still, the P2 differences were not linked to the BIAT D-score. It might be that these earlier differences reflect an early attention enhancement by the emotional congruency, preparing the participants to react faster. Considering re-entrant processing explanations (Pourtois et al., 2013), signaling from the amygdala regarding the emotional salience might have preceded task related signaling (as reported from intracranial recordings, see Pourtois et al., 2010). Eventually, the actual BIAT scores seem to be uniquely predicted by the later occurring LPP.

These results point to deliberate involvement in performing the doping BIAT. However, this does not imply that participants can easily choose how to respond to an IAT. First, the doping BIAT has been found to predict biochemical doping test results (Brand et al., 2014b). Further, when incentivized to fake doping attitudes, it has been found that participants were successfully changing their self-reported doping attitudes but not their BIAT scores (Wolff et al., 2015). It is thus concluded that IATs are less controllable and still more implicit than many other tests (De Houwer et al., 2009). It could be that although participants may be in general able to alter responses they are unwilling to change their IAT performance (e.g., they may be afraid to get caught or too exhausted to think about a successful strategy). This could also explain mixed results from faking studies (De Houwer et al., 2009).

Some limitations of the present research have to be mentioned. To avoid deviating too far from the original BIAT, we used a limited number of trials per condition (40 trials each). Therefore our scoring of the earlier components might be of limited accuracy (cf. Woodman, 2010). However, we used a peak area scoring for comparing differences on each component, which is found to be more reliable (Olvet and Hajcak, 2009) and thus are recommended in ERP research (Keil et al., 2014). Further, these results are found on the group level. The microvolt differences for both conditions were rather small. This corresponds to the overall doping attitude of our sample, which was only slightly negative (mean D-Score = −0.24). However, the correlations between the LPP and D-scores suggest, that the LPP differences might be underestimating the effects for the single subject with anti-doping attitudes. When only considering the two thirds of the sample with an anti-doping D-score, the LPP differences increase considerably [*t*_(26)_ = 3.76, *p* < 0.001, *d* = 0.40]. Future research should investigate if microvolt differences are bigger when participants hold a stronger attitude toward the target concept or attitudes toward a given concept are more homogenous. Finally, the potential inclusion of these results in meta-analyses warrants a note of caution. The doping attitudes investigated here were collected as baseline measures for two other studies that were concerned with investigating cerebral correlates of IAT faking (Schindler et al., 2015b; Wolff et al. in prep.). Thus, although these studies addressed different research questions, they rely on the same sample.

Summarizing the main findings, we could identify relatively early and presumably more automatic as well as late and more deliberate differences during BIAT completion. We had a sample of university students, which exhibited increased attention and faster reaction times for negative attitudes toward performance enhancing substances. Although, the enlarged occipital P2 for negative doping attitudes might be seen as a proof of the automaticity of activated associations during a doping BIAT, it is important to acknowledge that only the late positive potential was found to be associated with BIAT scores. This provides preliminary evidence that there is, next to an implicit component, a deliberate component in performing a BIAT.

In regard to doping, these results provide a first indication on the cerebral processes that are associated with the doping attitudes that are captured by the BIAT. The possibility to identify neural correlates of the BIAT score along with previous findings that these scores are associated with actual doping behavior (Brand et al., 2014b) and the relative robustness of such measures toward faking (Wolff et al., 2015) lends further weight to the importance of such implicit attitudes for doping research.

There are at least three reasons why we believe that our results are of interest to the broader concept of performance enhancing substance abuse (which incorporates NE, doping and other variants of drug instrumentalization). First, from a theoretical level doping and NE are similar as they imply using a substance as a means to performance enhancement. This similarity has been shown specifically for attitudes already: A domain-specific adaptation of a doping attitude questionnaire has been found to be a valid predictor of NE behavior (Wolff and Brand, 2013). Second, from a measurement perspective the stimuli that represent doping in the doping BIAT are rather unspecific. They contain pictures of syringes and pills. It would thus be worthwhile to assess if this doping BIAT can be reframed to the NE context. Third, social desirability has been found to affect responding in NE self-reports as well (Dietz et al., 2013a) and the doping BIAT has been found to be relatively robust toward such self-presentation efforts (Wolff et al., 2015). We encourage further research to develop such indirect measures for the NE domain as well. This doping BIAT represents a measure that has been heavily scrutinized from various angles by recent research (Brand et al., 2014a,b; Wolff et al., 2015; Schindler et al., 2015b) and might provide a good starting point for such endeavors in the NE domain. Understanding of the cerebral roots of attitudes toward performance enhancing substances will be an important step in further unraveling the psychology of NE.

### Conflict of interest statement

The authors declare that the research was conducted in the absence of any commercial or financial relationships that could be construed as a potential conflict of interest. The reviewer Sven Hoffmann and handling Editor declared their shared affiliation, and the handling Editor states that the process nevertheless met the standards of a fair and objective review.
